# Effects of VEGFR-3 phosphorylation inhibitor on lymph node metastasis in an orthotopic diffuse-type gastric carcinoma model

**DOI:** 10.1038/sj.bjc.6605296

**Published:** 2009-09-29

**Authors:** M Yashiro, O Shinto, K Nakamura, M Tendo, T Matsuoka, T Matsuzaki, R Kaizaki, M Ohira, A Miwa, K Hirakawa

**Affiliations:** 1 Department of Surgical Oncology, Osaka City University Graduate School of Medicine, 1-4-3 Asahi-machi, Abeno-ku, Osaka 545-8585, Japan; 2 Oncology Institute of Geriatrics and Medical Science, Osaka City University Graduate School of Medicine, 1-4-3 Asahi-machi, Abeno-ku, Osaka 545-8585, Japan; 3 Drug Discovery Research Laboratories, Kyowa Hakko Kirin Co., Ltd., Mishima, Shizuoka, Japan

**Keywords:** diffuse-type gastric carcinoma, lymph node metastasis, orthotopic gastric cancer model, phosphorylation inhibitor, vascular endothelial cell growth factor receptor-3

## Abstract

**Background:**

Vascular endothelial growth factor receptor-3 (VEGFR-3) signalling mediates lymphangiogenesis and lymphatic invasion; however, the effect of VEGFR-3 inhibition on the lymph node (LN) metastasis remains unclear. The aim of this study is to clarify the benefit of a VEGFR-3 inhibitor Ki23057 for LN metastasis.

**Methods:**

Ki23057 was administered orally to gastric cancer models created by orthotopic inoculation of diffuse-type gastric cancer cells, OCUM-2MLN. The effects of Ki23057 on lymphatic vessel invasion, lymphatic vessel density, and VEGFR-3 phosphorylation were examined by immunostaining or immunoblotting.

**Results:**

Ki23057 inhibited the autophosphorylation of VEGFR-3, with IC_50_ values of 4.3 nM in the cell-free kinase assay. Murine gastric cancer models created by the orthotopic inoculation of OCUM-2MLN cells showed the diffusely infiltrating growth and frequently developed LN metastasis. The oral administration of Ki23057 significantly (*P*<0.01) reduced the size of orthotopic tumours and the number of the metastatic LN in gastric cancer models. The degree of lymphatic invasion and lymphangiogenesis was significantly (*P*<0.05) lower in the gastric tumours treated by Ki23057. Ki23057 inhibited the phosphorylation of VEGFR-3 of lymphatic endothelial cells in gastric tumours.

**Conclusion:**

The inhibition of lymphangiogenesis targeting VEGFR-3 phosphorylation is a therapeutic strategy for inhibiting LN metastasis of diffuse-type gastric cancer.

The metastatic spread of tumour cells is responsible for the majority of cancer deaths. In most solid tumours (including gastric cancer), the spread of cancer cells through the lymphatics to regional lymph nodes (LN) is a frequent event in the initial process of cancer dissemination (Stacker *et al*, 2002a) and is an important prognostic indicator for patient outcome (Otsuji *et al*, 2004; Kunisaki *et al*, 2005; Yashiro *et al*, 2005). Nodal metastasis is considered to be dependent on the tumour-induced lymphangiogenesis or the invasion of preexisting lymphatic vessels (Clarijs *et al*, 2001). In experimental tumours, vascular endothelial growth factor (VEGF)-C and VEGF-D expressions induce lymphangiogenesis and correlate with lymphatic invasion and nodal metastasis (Amioka *et al*, 2002; Gombos *et al*, 2005; Shida *et al*, 2006). Of the three VEGF receptors (VEGF-Rs), VEGFR-3 signalling mediates lymphangiogenesis in tumours and appears to have a significant role in tumour metastasis through the lymphatics (Juttner *et al*, 2006). The signal through VEGFR-3 and VEGF-C is essential for the development of lymphatic vessels (Karkkainen *et al*, 2004). Targeting tumour lymphangiogenesis is considered to be an attractive strategy for the treatment of LN metastatic diseases because tumour-associated lymphatics are a key component of metastatic spread. However, the therapeutic strategy targeting VEGF/VEGFR-3 signalling to inhibit LN metastasis has been rare. Taken together, it remains unclear whether the inhibition of lymphangiogenesis targeting VEGFR-3 is a realistic therapeutic strategy for inhibiting tumour cell dissemination and the formation of nodal metastasis.

Ki23057, a newly developed tyrosine kinase inhibitor, competes with ATP for the binding site in the kinase and therefore blocks the autophosphorylation of VEGFR-3 and fibroblast growth factor receptor 2 (FGFR-2) (Shimizu *et al*, 2004; Nakamura *et al*, 2006). The characteristic clinical features of diffuse-type gastric carcinoma (DGC), a diffusely infiltrating type of gastric carcinoma also known as scirrhous gastric carcinoma, include a high frequency of metastasis to the LNs (Hippo *et al*, 2001; Nakazawa *et al*, 2003) and to the peritoneum (Yashiro *et al*, 1996; Takemura *et al*, 2004; Tendo *et al*, 2005). Although we currently reported the effects of Ki23057 on the peritoneal dissemination of DGC with an amplification of the activated *FGF-R2* (K-*sam*II) gene (Shimizu *et al*, 2004; Nakamura *et al*, 2006), no candidate agent as VEGFR-3 phosphorylation inhibitor has yet been proposed for the molecular target therapy of LN metastasis. The molecular controls of tumour-induced lymphangiogenesis through the VEGFR-3 signalling system may be targets for novel therapeutics designed to restrict LN metastasis. In this study, to examine the benefit of the VEGFR-3 inhibitor for DGC with LN metastasis, we explored the possibility of preventing metastasis using molecular target therapy of Ki23057.

## Materials and methods

### Phosphorylation inhibitor

Small-synthetic molecules that interrupt proliferation-related receptor tyrosine kinase (RTK) pathways, Ki23057 (Shimizu *et al*, 2004), was used in this study. Ki23057 was dissolved in distilled water, stored in a light-shielded container at 4 °C, and used within 5 days. For *in vivo* experiments, the agent dissolved in distilled water was orally administered. For *in vitro* experiments, the diluted Ki23057 was mixed at various concentrations with Dulbecco's modified Eagle's medium (DMEM; Nikken, Kyoto, Japan).

### Cell lines

The human gastric cancer cell line (OCUM-2MLN; Fujihara *et al*, 1998, 1999; Hippo *et al*, 2001) and the human umbilical vein endothelial cells (HUVECs) were used. OCUM-2MLN cell line was derived from DGC. OCUM-2MLN cells have a high LN metastatic potential in nude mice, as previously reported (Fujihara *et al*, 1998). OCUM-2MLN cells were cultured in medium consisted of DMEM with 10% heat-inactivated fetal bovine serum (FBS; Life Technologies, Grand Island, NY, USA). Human umbilical vein endothelial cells were purchased from Kurabo (Osaka, Japan) and cultured in endothelial cell growth medium 2 (HuMedia-EB2, Kurabo), which contains 2% FBS, EGF 10 ng ml^−1^, hydrocortisone 1 *μ*g ml^−1^, gentamicin 50 *μ*g ml^−1^, amphotericin B 50 ng ml^−1^, basic FGF 5 ng ml^−1^, and heparin 10 *μ*l ml^−1^.

### Animal models

The effects of Ki23057 administration on LN metastasis was examined using mouse models of DGC with LN metastases as described previously (Fujihara *et al*, 1998). Briefly, under ether anaesthesia, a median abdominal incision was made in 4-week-old female BALB/c nude mice (Clea Japan, Shizuoka, Japan). The stomach was exposed carefully and OCUM-2MLN cells (5 × 10^6^ cells per 100 *μ*l of DMEM) were inoculated orthotopically at the stomach wall of the antrum using 30-G needles (Handaya, Saitama, Japan). Beginning 7 days after tumour inoculation, medication was administered orally five times weekly for 2 weeks. As the elimination half-life (*t*_1/2_) of Ki23057 is 93 h on oral administration (Shimizu *et al*, 2004), orally five times weekly administration of Ki23057 contribute to efficacy. The mice were killed 3 weeks after tumour inoculation; gastric tumour size and total number and weight of metastatic LNs in tumours resected from Ki23057-treated animals (25 mg kg^−1^ day^−1^; *n*=8) were compared with those in tumours resected from vehicle control animals (*n*=8). All experiments with nude mice were performed in accordance with the guidelines approved by the United Kingdom Coordinating Committee on Cancer Research (1998).

### Immunohistochemical techniques

Hematoxylin and eosin staining, Masson trichrome staining, and the immunohistochemical determination of lymphatic vessel endothelial hyaluronan receptor-1 (LYVE-1), CD31, VEGF-C, and phosphorylation of VEGFR-3 were examined using orthotopic tumours between the control and Ki23057-treated mice. Briefly, paraffin-embedded sections were cut at 5 *μ*m. After the deparaffinization and heating in Target Retrieval Solution (Dako, Carpinteria, CA, USA), the sections were incubated with LYVE-1 (2 *μ*g ml^−1^; cat no. 103-PA50AG) (RELIATech, Braunschweig, Germany), anti-CD31 antibody (sc-1506; Santa Cruz Biotechnology, Santa Cruz, CA, USA), anti-VEGF-C antibody (Zymed, South San Francisco, CA, USA), anti-Flt-4 antibody (BD Pharmingen, San Diego, CA, USA), or phosphotyrosine (Zymed), and were treated with a secondary antibody.

### Quantitative analysis of lymphatic vessel and microvessel

Lymphatic vessel invasion (LVI) and lymphatic vessel density (LVD) were determined by the hotspot method as previously described (Gombos *et al*, 2005). Briefly, slides were scanned at low power, and intratumoural and peri-tumoural areas with the highest density of LYVE-1-positive vessels were identified. Lymphatic vessel endothelial hyaluronan receptor-1 is used to evaluate intratumoural lymphatic vessels in solid tumours. Lymphatic vessel invasion or LVD was determined by counting the number of LYVE-1-positive vessels with or without cancer emboli in four high-power fields ( × 100) in areas of the highest lymphatic vessel as ‘hot spots’. Microvessel densities (MVDs) were evaluated using immunohistochemical detection of CD31. Four fields per tumour were scored for tumour microvessel elements at × 200 magnification, and the number of microvessel elements per field was calculated as MVD. Areas of necrosis were avoided.

### Cellular and cell-free kinase assays

The proliferation of HUVECs in the presence and absence of VEGF was evaluated using WST-8 colorimetric assay. Human umbilical vein endothelial cells were seeded at 2 × 10^3^ cells per well on a 96-well plate in the HuMedia-EB2 medium. Each well was treated with Ki23057 at various concentrations (0, 10, 30, 100, and 300 ng ml^−1^). VEGF (10 ng ml^−1^) was subsequently added to each well and the plate was incubated for 72 h. The live cell count was assayed using Cell Counting Kit-8 (Dojin Laboratories, Kumamoto, Japan) according to the instructions provided by the manufacturer, and the absorbance of each well was measured at a test wavelength of 450 nm and a reference wavelength of 630 nm with a microtiter plate reader. Six replicate wells were used for each drug concentration, and the testing was carried out independently for thrice.

The ability of Ki23057 to inhibit VEGFR-3 phosphorylation was evaluated using HUVECs that express the RTK of VEGFR-3, as follows. Cells were harvested and plated onto the collagen I-coated dishes and serum-starved in EBM-2 supplemented 0.5% FBS for 16 h. After the addition of Ki23057, cells were incubated for 1 h and then stimulated with 100 ng ml^−1^ recombinant rat VEGF-C (RELIATech GmbH, Braunschweig, Germany) for 20 min at 37 °C. After cell lysis, VEGFR-3 was immunoprecipitated with anti-Flt-4 antibodies (Santa Cruz Biotechnology) and subjected to immunoblotting with phosphotyrosine (Nakamura *et al*, 2004).

The amount of phosphorylated and total protein for VEGFR-3 in gastric tumour tissue was determined as follows. Briefly, tumour-bearing mice were treated with either a vehicle control or Ki23057. The mice were killed at 3 weeks after orthotopic inoculation. Resected orthotopic tumours were snap-frozen on dry ice and lysed in lysis buffer. The anti-Flt-4 antibodies react with mouse VEGFR-3 (BD Pharmingen, San Diego, CA, USA), and were immunoprecipitated and subjected to immunoblotting with phosphotyrosine (Zymed). The extent of phosphorylation in orthotopic tumours resected from Ki23057-treated animals was compared with that in tumours resected from vehicle control animals.

### Statistical analysis

The quantitative ratios of different groups were compared using Student's *t*-test. A difference was considered significant when the *P*-value was ⩽0.05.

## Results

### Effect of Ki23057 on the VEGFR-3 phosphorylation *in vitro*

**Figure 1:**
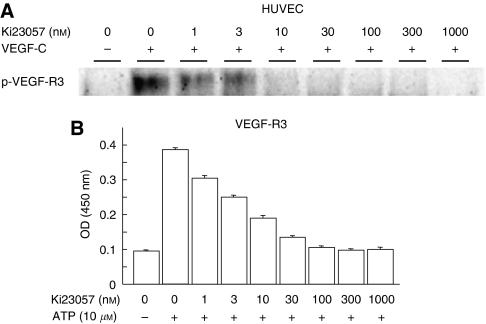
Effect of Ki23057 on VEGFR-3 phosphorylation. (**A**) The ability of Ki23057 to inhibit VEGFR-3 phosphorylation was evaluated using human umbilical vein endothelial cells (HUVECs) that express the RTK of VEGFR-3. Ki23057 inhibited the tyrosine phosphorylation of VEGFR-3 with IC_50_ values of 4.3 nM. (**B**) For cellular assay, HUVECs were incubated with serial concentrations of Ki23057. Ki23057 inhibited the growth of HUVEC with VEGFR-3 activated by VEGF-C. Ki23057 inhibited the autophosphorylation of VEGFR-3 at a dose-dependent manner. IC_50_ kinase value was estimated as 37 nM.

In biochemical assays using HUVECs that express the RTK of VEGFR-3, Ki23057 exhibited competitive inhibition against VEGFR-3 with an IC_50_ kinase value of 4.3 nM (Figure 1A). Ki23057 inhibited the autophosphorylation of VEGFR-3 with IC_50_ values of 37 nM in the cellular assay (Figure 1B).

### Effect of Ki23057 administration on DGC with LN metastasis *in vivo*

**Figure 2:**
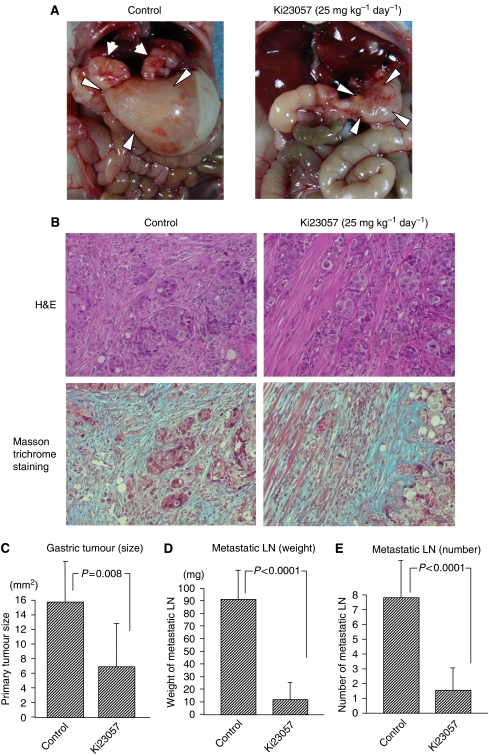
Effects of Ki23057 in diffuse-type gastric carcinoma with lymph node metastasis. (**A**) Macroscopic findings of gastric tumours with lymph node metastasis. The orthotopic inoculation showed diffuse-type gastric tumours (arrowheads). The area of the orthotopically transplanted tumours in Ki23057-treated mouse (right panel) was less than that of the control (left panel). Large LN metastases (arrows) in control mice were recognised around the primary gastric tumour. Lymph node metastases in Ki23057-treated mice were fewer in number and smaller than in controls. No toxicity or body weight loss was observed in any of the groups. (**B**) H&E staining and Masson trichrome staining. Orthotopic tumours of OCUM-2MLN cells showed extensive fibrosis with the occasional presence of poorly differentiated adenocarcinoma cells that resembled DGC. Extensive stromal fibrosis was also found in OCUM-2MLN gastric tumours treated by Ki23057. No remarkable difference was found in the histological findings of orthotopic carcinomas between the control and Ki23057-treated mice by H&E staining. (**C**) The mean areas of the orthotopically transplanted tumours in the control and Ki23057-treated mice were 15 and 6 mm^2^, respectively. Primary gastric tumours in mice receiving Ki23057 (25 mg kg^−1^ day^−1^) were significantly (*P*=0.008) smaller than those in controls. (**D** and **E**) The mean weights of metastatic LN in the control and Ki23057-treated mice were 91 and 11 mg, respectively. The mean number of metastatic LN in the control and Ki23057-treated mice were 7.8 and 1.5, respectively. Ki23057 significantly decreased both the mean weight (*P*<0.0001) and the mean number (*P*<0.0001) of involved nodes.

Macroscopic findings of gastric tumours by the orthotopic inoculation of OCUM-2MLN cells showed the diffusely infiltrating growth resulting in thickening of the gastric wall accompanied by pyloric stenosis, which resembled human DGC. The involvement of numerous LNs was detected around the stomach, which was similar to the development of human DGC with LN metastasis (Figure 2A, left panel). The area of the orthotopically transplanted tumours in mice treated with 25 mg kg^−1^ day^−1^ of Ki23057 (Figure 2A, right panel) was less than that of the control. The nodal metastases in Ki23057-treated mouse were fewer and smaller, in comparison with the control. Development of a gastric tumour was found in eight out of eight (100%) control mice, whereas that was five out of eight (63%) Ki23057-treated mice. Orthotopic tumours of OCUM-2MLN cells showed extensive fibrosis with the occasional presence of poorly differentiated adenocarcinoma cells that resembles the characteristic histological findings of human DGC by H&E staining, in both the control and Ki23057-treated mice (Figure 2B). Masson trichrome staining showed blue-colored multiple fibrosis in the both orthotopic tumours, whereas no fibrotic change was found among the two groups (Figure 2B). The primary tumours in mice receiving Ki23057 were significantly smaller than those in the control mice (Figure 2C). The weight and the number of LN metastases in mice receiving Ki23057 were significantly lower than those in the control mice (Figure 2D and E). No mice died during the observation period. Neither weight loss nor any skin abnormalities were associated with the administration of Ki23057. We added these comments to the results.

### Effect of Ki23057 administration on LVD of DGC *in vivo*

**Figure 3:**
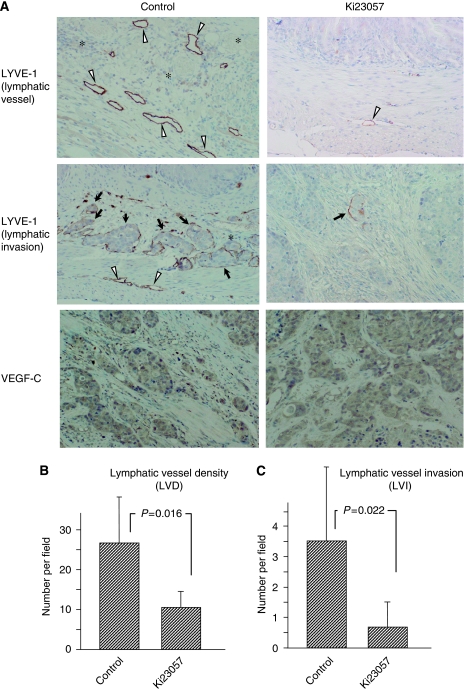
Immunohistochemical expression of LYVE-1 and VEGF-C in orthotopic tumours. (**A**) Orthotopically planted OCUM-2MLN cells frequently invade into lymphatic vessels. The number of lymphatic vessels at the invasive edge of tumours was fewer in mice treated by Ki23057, compared with the control. The number of lymphatic invasion was fewer in the gastric tumours treated by Ki23057, compared with the control. Lymphatic vessels located at the invasive edge of tumours were often enlarged and dilated. Expression level of VEGF-C was not different between the two groups. The LYVE-1 antibody stains lymphatic vessels (arrowheads) and highlights lymphatic invasion (arrows). Many lymphatic vessels were found at the invasive edge of tumours. The LYVE-1 antibody-stained vessels devoid of red blood cells corresponded to lymph vessels, whereas blood vessels were not stained (asterisks). Single-stained endothelial cells were excluded. (**B**) Lymphatic vessel density (LVD) was determined by LYVE-1-positive vessels without cancer emboli. Respective mean LVD in control and Ki23057 mice respectively were 26.5 and 10.4. The mean LVD was significantly decreased by Ki23057. (**C**) Lymphatic vessel endothelial hyaluronan receptor-1 immunostaining highlighted the presence of lymphatic invasion. Lymphatic vessel invasion was identified as the presence of cancer emboli within the channels lined by LYVE-1-positive vessels. Respective mean LVI (lymphatic vessel invasion) in control and Ki23057 mice were 3.5 and 0.7. The LVI was significantly decreased by Ki23057. Lymphatic vessel invasion or LVD was determined by counting the number of LYVE-1-positive vessels with or without cancer emboli in four high-power fields ( × 100) in areas of the highest lymphatic vessel as ‘hot spots’.

Vessels with a lumen that stained with LYVE-1 antibody were considered lymphatic vessels. Lymphatic vessel density was determined by LYVE-1-positive vessels without cancer emboli. Lymphatic vessel invasion was identified as the presence of cancer emboli within the channels lined by LYVE-1-positive vessels. The number of lymphatic vessels and lymphatic invasion was fewer in mice treated by Ki23057, compared with the control. VEGF-C expression was heterogeneous within the tumours; however, the expression levels of VEGF-C were not different between the control and Ki23057-treated mice (Figure 3A). In comparison with the control tumours, LVD was significantly (*P*=0.016) less in the gastric tumours treated by Ki23057 (Figure 3B). The extent of LVI was significantly (*P*=0.022) less in the gastric tumours treated by Ki23057 in comparison with that in the control (Figure 3C).

### Effects of Ki23057 on the phosphorylation of VEGFR-3 *in vivo*

**Figure 4:**
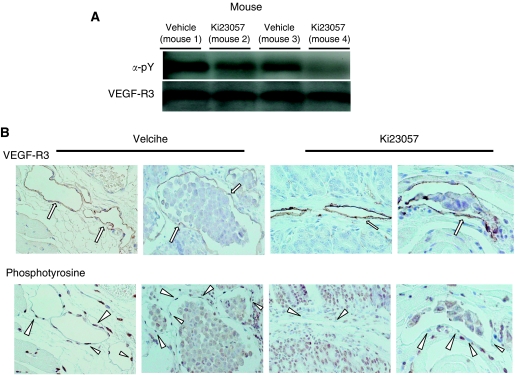
Inhibitory effect of Ki23057 on VEGFR-3 phosphorylation. (**A**) The inhibitory effect of Ki23057 on the phosphorylation of VEGFR-3 in gastric tumour specimens. VEGFR-3 phosphorylation was suppressed in the tumours of the Ki23057-treated mice (mouse 2 and mouse 4), in comparison with that in the vehicle mice (mouse 1 and mouse 3). (**B**) The effects of Ki23057 on VEGFR-3 phosphorylation levels on lymphatic endothelial cells. To assess the phosphorylation of VEGFR-3 in tumour tissue, the anti-Flt-4 antibody reacts with mouse VEGFR-3 and phosphotyrosine was examined. Tumours treated by Ki23057 showed an apparent attenuation of phosphorylation (arrows) on the lymphatic endothelial cells of VEGFR-3 staining (arrowheads), in comparison with that in vehicle mice.

To assess phosphorylation in tumour cells, tumour-bearing mice were treated with vehicle control or a single dose of Ki23057. Figure 4A shows the inhibitory effect of Ki23057 on the phosphorylation of gastric tumour specimens. VEGFR-3 phosphorylation was suppressed in the tumours of Ki23057-treated mice (mouse 2 and mouse 4) in comparison with that in the vehicle mice (mouse 1 and mouse 3). The tumours from the control mice that received vehicle only showed the strong staining of phosphotyrosine on the lymphatic endothelial cells of VEGFR-3 staining. In contrast, the tumours from mice that received Ki23057 showed weak staining of phosphotyrosine on the lymphatic endothelium (Figure 4B).

## Discussion

A few gastric cancer models with LN metastasis have been reported (Yamaguchi *et al*, 2000; Yonemura *et al*, 2000; Takemura *et al*, 2004). In this study, the *in vivo* gastric cancer model with OCUM-2MLN cells showed extensive fibrosis with the occasional presence of diffuse-type adenocarcinoma cells, resulting in thickening and stiffening of the gastric wall. In addition, the orthotopically planted OCUM-2MLN cells frequently invade into lymphatic vessels and displayed frequent LN metastasis. These findings suggest that this model resembled human DGC and was useful for the evaluation of therapies for primary gastric tumour and LN metastasis.

The metastatic spread of tumour cells causes the vast majority of cancer deaths. Clinical and pathological evidence indicated that the metastatic spread of tumours through lymphatic vessels to local/regional LNs is an early event in metastatic disease for many solid human tumours (Stacker *et al*, 2002b). Lymph node metastasis is an important prognostic indicator for patient with DGC (Otsuji *et al*, 2004; Kunisaki *et al*, 2005). Tumour cells can escape from the primary site by entering existing vessels or new vessels actively recruited into the primary tumour (Stacker *et al*, 2002b). Therefore, the VEGF-C/VEGFR-3 signalling system for lymphangiogenesis might constitute a potential new target for the development of anti-cancer therapeutics (Stacker *et al*, 2002b). In this study, the LN metastases in mice treated with Ki23057 were significantly smaller and fewer in comparison with the metastases detected in the controls. The LVD values in the gastric tumours were examined using LYVE-1 staining. The degree of lymphatic invasion and the number of lymphatic vessels were significantly fewer in the tumours treated by Ki23057. VEGFR-3 signalling mediates lymphangiogenesis in tumours and would have a significant role in tumour metastasis through the lymphatics (Kitadai *et al*, 2005; Juttner *et al*, 2006). Ki23057 showed considerable inhibitory activity against autophosphorylation of VEGFR-3 *in vitro* and *in vivo*; the IC_50_ for VEGFR-3 is 4.3 nM in the cellular assay and 5.4 nM in the cell-free kinase assay. Immunohistochemical studies also showed that the oral administration of Ki23057 showed the inhibitory effect of VEGFR-3 phosphorylation for the lymph endothelial cells in the tumours of the mice. In contrast, the expression level of VEGF-C was not affected by Ki23057. These findings suggest that Ki23057 is a promising molecular targeting agent for LN metastasis working as a VEGFR-3 inhibitor against lymphangiogenesis in DGC. The VEGFR-3 signalling system might be one of attractive targets for antilymphangiogenic therapeutics designed to restrict LN metastasis.

It is still controversial whether nodal metastasis is dependent on tumour-induced lymphangiogenesis or the invasion of preexisting lymphatic vessels (Clarijs *et al*, 2001). Our findings suggest that LN metastasis is dependent on tumour-induced lymphangiogenesis and indicate that the inhibition of lymphangiogenesis targeting VEGFR-3 is a realistic therapeutic strategy for inhibiting LN metastasis. In contrast, immunohistochemical study by CD31 showed that Ki23057 treatment resulted in no inhibition of tumour MVD, which indicated that Ki23057 did not affect angiogenesis within the primary tumours of DGC. Diffuse-type gastric carcinoma has been reported to show the hypo-vascularity and low VEGF expression, and the vessel count was not correlated with the presence of endothelial VEGFR-2 in DGC (Takahashi *et al*, 1996). These findings might explain the reason for the ineffectiveness of Ki23057 on the tumour MVD by CD31 staining in the DGC tumour vasculature.

The oral administration of Ki23057 resulted in a reduction of the gastric tumour size. We have previously reported that proliferation of DGC cells was stimulated by FGF-7, which is a ligand of FGFR-2, but not by VEGF. Ki23057 blocks the autophosphorylation of fibroblast growth factor receptor 2 and VEGFR-3 (Shimizu *et al*, 2004; Nakamura *et al*, 2006). Ki23057 might decrease the growth of DGC tumour by inhibiting the FGFR-2 signalling pathway.

In conclusion, the inhibition of lymphangiogenesis targeting VEGFR-3 is a realistic therapeutic strategy for inhibiting LN metastasis. The autophosphorylation inhibitor against VEGFR-3, Ki23057, might therefore be useful for the treatment of LN metastasis of DGC.
